# P12 Commissioning for Quality and Innovation (CQUIN) schemes—revisiting urinary tract infection (UTI) management. What worked? What lasted? What next?

**DOI:** 10.1093/jacamr/dlac133.016

**Published:** 2023-01-19

**Authors:** Mandy Slatter, Valentin Aprilia, Rebecca Boyd, Teresa Chan

**Affiliations:** Royal United Hospitals Bath NHS Trust, UK; Department of Life Sciences, University of Bath, UK; Department of Life Sciences, University of Bath, UK; Department of Life Sciences, University of Bath, UK

## Abstract

**Background:**

UK CQUIN schemes encourage an improvement focus on a specific area of care. In 2019, CQUIN CCG1a: Improving the management of lower UTI in older people^1^ was adopted at the RUH resulting in increased alignment with UK guidance on diagnosis and treatment.^2^ Our aim was to investigate whether this improvement was sustained two years later. This would help inform quality improvement interventions prior to adoption of the 2022 CQUIN, CCG2: Appropriate antibiotic prescribing for UTI in adults aged 16+.^3^

**Objectives:**

Service evaluation of the UTI pathway including compliance with two of the CQUIN care processes for UTI diagnosis in patients age 65+ presenting to ED (not admitted): (i) diagnosis excludes use of urine dipstick in people aged 65+; and (ii) urine sample sent to microbiology as per UK guidance.^2^

**Methods:**

A search of the electronic patient record for key terms (Table 1) identified 6076 ED attendances for patients age 65+ between 1 August and 31 October 2021 of which 40 were identified with a primary diagnosis of UTI not requiring hospital admission. Paramedic, ED and Urgent Treatment Centre notes (paper and electronic) were reviewed in detail and information gathered regarding presence/absence of UTI symptoms aligned to diagnostic guidelines;^2^ presence/absence of urine dipstick test; and presence/absence of urine sample for culture and susceptibility testing. Findings were compared with identical trust data for patients (admitted and non-admitted) obtained during the 2019 CQUIN: Q1 April–June; Q2 July–September; Q3 October–December. During this period improvement interventions were implemented.

**Results:**

See Table 2.

**Conclusions:**

Following intensive staff education improved practice regarding urine dipstick testing and appropriate urine sampling in elderly patients with possible UTI was observed during the 2019 CQUIN period. Two years later this improvement had not been sustained. When planning interventions during the 2022 CQUIN,^3^ consideration should be given to a bundle of interventions including education, data feedback and systems improvement, for example, computerized decision support systems (CDSS) to embed sustained change.

**Table 1.** Key terms used to search electronic patient record when identifying eligible patients

Search terms	Search fields
Urin, UTI, pyelonephritis, cystitis, urosepsis, CAUTI, AKI, Acute Kidney Injury, Flank pain	Reason for visit, Chief Complaint, Presenting Complaint, ECDS

**Table 2.** Percentage compliance with CQUIN care processes for UTI diagnosis in patients age 65+

Data collection period	No. of patients	Patients with urine dipstick, % (target 0%)	Patients with documented symptoms and urine sample, % (target 100%)	Patients with no documented symptoms and urine sample, % (target 0%)	Urine sampling decision aligned with UK guideline,^2^%
2019 CQUIN Q1	100	20	89	95	72
2019 CQUIN Q2	100	3	93	79	83
2019 CQUIN Q3	100	6	93	60	90
COVID pandemic					
ȃPre 2022 CQUIN	40	68	60	47	58
